# Implementation of a Mindfulness-Based Crisis Intervention for Frontline Healthcare Workers During the COVID-19 Outbreak in a Public General Hospital in Madrid, Spain

**DOI:** 10.3389/fpsyt.2020.562578

**Published:** 2020-10-30

**Authors:** Beatriz Rodriguez-Vega, Ángela Palao, Ainoa Muñoz-Sanjose, Marta Torrijos, Pablo Aguirre, Arancha Fernández, Blanca Amador, Cristina Rocamora, Laura Blanco, Jesús Marti-Esquitino, Aránzazu Ortiz-Villalobos, Mónica Alonso-Sañudo, Susana Cebolla, Javier Curto, Rosa Villanueva, María-Jesús de-la-Iglesia, Diego Carracedo, Carlos Casado, Emma Vidal, Daniel Trigo, Noelia Iglesias, Diana Cabañas, Loreto Mellado, Daniel García, Consuelo Fernández-Encinas, Rubén Navarro, Roberto Mediavilla, María-Paz Vidal-Villegas, María-Fe Bravo-Ortiz, Carmen Bayón

**Affiliations:** ^1^Psychiatry, Clinical Psychology and Mental Health Department, La Paz University Hospital, Madrid, Spain; ^2^Hospital La Paz Institute for Health Research (IdiPAZ), Madrid, Spain; ^3^Autonomous University of Madrid (UAM), Madrid, Spain

**Keywords:** mindfulness, brief mindfulness-based intervention, compassion, stress, COVID-19, healthcare workers, implementation, general hospital

## Abstract

**Introduction:** The COVID-19 outbreak is having an impact on the well-being of healthcare workers. Mindfulness-based interventions have shown effectiveness in reducing stress and fostering resilience and recovery in healthcare workers. There are no studies examining the feasibility of brief mindfulness-based interventions during the COVID-19 outbreak.

**Materials and Methods:** This is an exploratory study with a post intervention assessment. We describe an on-site brief mindfulness intervention and evaluate its helpfulness, safety, and feasibility.

**Results:** One thousand out of 7,000 (14%) healthcare workers from La Paz University Hospital in Madrid (Spain) participated in at least one session. One hundred and fifty out of 1,000 (15%) participants filled out a self-report questionnaire evaluating the helpfulness of the intervention for on-site stress reduction. Ninety two subjects (61%) participated in more than one session. Most of the participants were women (80%) with a mean age of 38.6 years. Almost half of the sample were nurses (46%). Sessions were perceived as being helpful with a mean rating of 8.4 on a scale from 0 to 10. Only 3 people (2%) reported a minor adverse effect (increased anxiety or dizziness).

**Discussion:** Our data supports the utility, safety and feasibility of an on-site, brief mindfulness-based intervention designed to reduce stress for frontline health workers during a crisis. There is a need to continue testing this type of interventions, and to integrate emotion regulation strategies as an essential part of health workers' general training.

**Clinical Trial Registration number:** NCT04555005.

## Introduction

The pandemic caused by the SARS-CoV-2 poses a major challenge for national health systems around the globe. Along with Italy, Spain was one of the European epicenters of the pandemic, with more than 220,000 people infected and over 25,000 dead by May 15th 2020, the core period of the pandemic in Madrid (Spain) until date (1). Currently (by August 10th) 322,980 people have been infected and 28,576 have died (2). Almost one third of the people infected were diagnosed in the region of Madrid, where more than 8,000 people died from February 25th to May 15th (8,464 to August 10th). Hospitals had to change their structure almost entirely in order to effectively respond to the emergency.

La Paz University Hospital is a public, general hospital in Madrid that provides healthcare to a catchment area of over 500,000 people. Around 3,000 patients infected with SARS-CoV-2 have been treated in this Hospital by May 15th (3). Since the beginning of March 2020, most of its units were converted to COVID-19 wards, non-emergency surgeries were canceled, and beds in intensive care units (ICUs) were quadrupled. Additionally, many professionals from different specialties were deployed to the frontline. This entailed working with unknown colleagues in novel settings where safety and trust are critical.

Healthcare workers have been a vulnerable population exposed to close contact with infected patients, to an excessive workload and to experiences of physical exhaustion, fear, emotional disturbance, and dysregulation of sleep patterns during the COVID-19 pandemic (4–8). Many studies have been conducted in China during the outbreak, and the results reveal high rates of anxiety and depressive symptoms, sleep problems and psychological distress in more than 70% of the surveyed samples (4, 9). In addition, follow-up studies show that psychological effects may persist long after the outbreak. Wong et al. found that 3 years after the 2003 SARS outbreak, 23% of healthcare workers reported moderate to greater depressive symptoms (10). This outbreak is also putting healthcare workers into ethical and moral dilemmas. They have to make decisions that may include how to allocate scant resources to equally needful patients, how to balance their own physical and mental health needs with those of the patients and how to align their commitment to help patients with their willingness to be with family and friends (11). In light of this situation, the Word Health Organization has made recommendations for identification and management of physical, mental health and psychosocial well-being in healthcare workers (12, 13).

Previous studies outline the importance of safeguarding the morale and mental health of healthcare professionals as this can influence the success of healthcare delivery (14, 15). Stigma and abandonment have been reported across various outbreaks despite differences in culture, education levels and available healthcare services (16, 17). Besides physical recommendations that may help reduce psychiatric symptoms, studies emphasize as coping strategies the support from colleagues and sustained engagement with updated, reliable information about the outbreak (18).

Our Mental Health Team participated in the Ebola Health Emergency in Madrid (Spain) in 2014. A total of 100 people, most of them healthcare workers, were attended (19). One emphasized conclusion was that the mental health team should be involved during the emergency from initial stages providing training in emotion regulation techniques for the rest of professionals.

Mindfulness is the ability to pay attention to the present moment in an intentional, non-judgmental way (20). Mindfulness-based programs have shown efficacy in reducing stress (21) and increasing quality of life and self-compassion in healthcare professionals (22). Gilmartin et al. conclude in a systematic literature review that brief mindfulness interventions (lasting 5–20 min once a day) may be effective in improving healthcare provider's well-being and decreasing levels of anxiety and stress (23). Furthermore, mindfulness training is associated with emotion regulation, fostering well-being and resilience and promoting switching from a state of automatic pilot to one of cognitive awareness, enabling a more thoughtful approach to clinical decision-making (24–26). Other studies outline the importance of self-compassion because of its positive association with happiness and recommend to include specific self-compassion components in future programs aimed at enhancing well-being in healthcare workers (27, 28).

Mindfulness can certainly be a “pathway to resilience and recovery” during the COVID-19 pandemic (29). One of the actions taken by La Paz University Hospital's Mental Health Team was developing a brief Mindfulness-based intervention for frontline healthcare workers to train emotion regulation. Following the recommendations given by some authors with experience in mindfulness training (24, 30), the intervention was conceived as a brief experience (between 5 and 10 min), delivered on-site (at COVID-19 wards) and repeatedly (twice a day, 7 days a week), during 7 weeks.

The aim of the present study is to describe an on-site, brief mindfulness-based crisis intervention and explore its feasibility, helpfulness and safety for frontline healthcare workers in the midst of the COVID-19 storm.

## Materials and Methods

This study was developed as an exploratory research design with a post-intervention assessment.

From the beginning of the emergency, at least two members of the Mental Health Team went to the places where frontline health professionals were working (emergency department, ICUs, COVID-19 wards) and offered the intervention on-site.

The Ethics Review Board approved the study and concluded that due to the emergency situation and the fact that such type of intervention was delivered as routine care at our hospital, participants' consent was not required. Trial registration number: NCT04555005.

### Measures

An anonymous, short self-report questionnaire was designed *ad hoc* to collect the following variables: age, gender, profession, workplace, session attendance, and perceived helpfulness in reducing current stress (ranked on a 0–10 point visual analog scale). Subjects filled out the questionnaire right after the session.

We collected the following data as indicators of the utility, safety and feasibility of the implementation of this intervention:

Utility:

- Mean “perceived helpfulness in reducing current stress.”

Safety:

- Number and % of participants who reported any kind of adverse event.

Feasibility:

- Number and % of professionals who attended at least one session, out of the total number of health care workers of the hospital.
- Number of sessions that were held in COVID-19 wards between March 10th and April 26th.
- Number and % of participants who filled out the survey out of the total number of professionals who attended at least one session.
- Number and % of professionals who attended more than one session.

### Intervention

The intervention consisted of 5–10 min of mindfulness practices delivered twice daily by experienced psychiatrists, psychologists, and mental health nurses. They were supervised by certified mindfulness trainers. The intervention was presented to each new team with an introduction as a justification of the action based on: (1) The importance of self-care, as professionals are the most valuable means the system has to deal with the crisis. There is no care for others if there is no care for oneself; (2) Placing mind training and emotion regulation at the same level of importance as the dressing and undressing of the personal protective equipment; and (3) The need to build an inner space of calm in the midst of the storm, from which successful actions can be taken. This explanatory introduction seems a key element to improve the acceptability and the adherence to the intervention. Three elements were trained in each session: (1) Focused attention through the invitation to kindly rest attention on a specific anchor, such as breathing, parts of the body like hands or feet, or the surrounding sounds; (2) Conscious movements through soft hatta yoga stretching exercises which were done standing or sitting, adapted to any physical condition; and (3) Compassion, through kind and inviting language and attitude, *via* specific sentences and gestures which invite to care for oneself (i.e., placing one or both hands on the chest). Participants were invited to recognize and accept without judgment any emotion, thought and body sensation that arose during the practice.

Sessions were characterized by being proactive, on-site, flexible, repetitive, generating an internal pause and place of empowerment. Flexibility was manifested in the order in which each element was presented and the duration, which could be changed depending on the context and the level of energy or concern of each team at each moment. For example, if we felt that the team was highly aroused, we started with a set of conscious movements. The aim was to recognize the tension and restlessness present at that particular moment and to invite, through the practice, to focus the attention to the present moment in a kind and compassionate way.

## Results

More than 3,000 sessions were held in COVID-19 wards between March 10th and April 26th, the core period of the pandemic in Madrid (Spain) until date. Any worker of La Paz University Hospital could participate. One thousand out of 7,000 healthcare professionals (e.g., physicians, nurses, social workers, physical therapists, technicians, cleaning staff) attended at least one of the sessions. Therefore, the initial enrollment rate was of 14%. Table 1 shows, utility, safety and feasibility outcomes.

**Table 1:** Utility, safety and feasibility outcomes.

Utility, Mean (SD)	
Indicator 1^a^	8.4 (1.55)
Safety, *n* (%)	
Indicator 2^b^	3/150 (2%)
Feasibility	
Indicator 3, *n* (%)^c^	1000/7000 (14%)
Indicator 4, (number of sessions)^d^	>3000
Indicator 5, *n* (%)^f^	150/1000 (15%)
Indicator 6, *n* (%)^e^	92/150 (61%)

*Note*.

a *Indicator 1: Mean “perceived helpfulness in reducing current stress.”*

b *Indicator 2: Number and % of participants who reported any kind of adverse event, out of the total number of participants who filled out the survey*.

c *Indicator 3: Number and % of professionals who attended at least one session, out of the total number of healthcare workers at the hospital*.

d *Indicator 4: Number of sessions that were held in COVID-19 wards between March 10th and April 26th*.

e *Indicator 5: Number and % of participants who filled out the survey out of the total number of professionals who attended at least one session*.

f *Indicator 6: Number and % of professionals who attended more than one session*.

The rate of survey completion collected for 3 days (from April 13th to April 15th) was 15% (150/1,000). Demographic characteristics of the participants are shown in Table 2, while Table 3 shows data of the intervention. Most interventions were carried out in Intensive Care Units (23%), COVID-19 Medical Units (38%), and the Emergency Department (22%). Ninety two participants out of 150 (61%) attended more than one session.

**Table 2:** Characteristics of the participants.

Age (years), Mean (SD)	38.6 (12.3)
Sex, *n* (%)^a^
Women	119 (79.9)
Men	30 (20.1)
Profession, *n* (%)
Nurse	52 (46)
Nursing assistant	35 (31)
Orderly	11 (9.7)
Nursing Resident	1 (0.9)
Medical Resident	2 (1.8)
Physician	8 (7.1)
Cleaning Staff	2 (1.8)
Technician	2 (1.8)

a *No missing values*.

**Table 3:** Characteristics of the intervention.

Participants per session, M (SD)	7.43 (2.57)
Location, *n* (%)^a^	
ICUs	32 (22.7)
Emergency Department	31 (22)
Medical Unit	54 (38.3)
Physiotherapy Unit	10 (7.1)
Radio-Oncology Unit	10 (7.1)
Radiology Unit	2 (1.4)
Central Services	2 (1.4)
Times of participation in a session during the crisis^b^	
1	33 (26.4)
2–5	67 (53.6)
>5	25 (20)

a *Data of valid percentage has been used in all cases*.

Participants perceived the intervention as being helpful for reducing stress with a mean rating of 8.4 on a scale from 0 to 10. There was no significant statistical difference (*t* = −0.599, α > 0.05) on the perceived helpfulness between those participants who attended just one session (mean = 8.4; SD = 1.7) and those who attended more than one session (mean = 8.6; SD = 1.3).

Mild adverse effects were found in 3 participants (2%) who reported dizziness and increased anxiety after the session.

## Discussion

This study explores whether an on-site mindfulness-based crisis intervention designed for stress reduction for frontline healthcare professionals could be implemented on acute health wards of a public, general hospital during the outbreak of the COVID-19 pandemic in Madrid and shows data of feasibility, utility and safety of the intervention.

One core issue in this research is that the team developed and implemented the intervention in a short period of time and under extremely adverse circumstances (scarcity of protection materials and equipment, professional fatigue due to long shifts, hospital wards closed due to the infection, etc.). In this context, the initial enrollment was of one in every seven workers (14% participation rate). La Paz University Hospital has more than 7,000 workers but we could not access all of them probably due to factors such as fatigue, shifts, sick leaves, lack of time (the intervention was offered during working hours), among others. Moreover, not all of the 7,000 professionals were frontline health workers.

We collected questionnaires for 3 days only. Results of the 150 people (15% of the global participation) show that participants perceived the intervention as being helpful for reducing stress with a mean rating of 8.4 on a scale from 0 to 10, with a continuous participation rate over 61%, and few mild adverse effects (2%). We are aware of the fact that there may be some bias among people who completed the survey -e.g., those who found it particularly helpful may have been more likely to respond-. In addition, the rate of participation (14%) might reflect the bandwidth of the research team to only be able to reach a portion of providers. Although certainly the benchmark for determining feasibility may be lower during a pandemic, there is no established cut-off for what that benchmark would be. Taking these limitations into account our data suggest feasibility and good utility and safety outcomes during a pandemic.

Randomized controlled trials are the best study design to test the comparative effect of an intervention. This was far from the objective of the current research. We cannot answer the questions “was this intervention effective?” or “was there any change after the intervention?” The intervention was rated by health workers as very helpful with no differences in the ratings between those who attended one session and those who attended more sessions. This finding is similar to Gilmartin's conclusion (24). To our knowledge, there are no studies that evaluate the implementation of a brief mindfulness-based intervention during a crisis.

The acceptability of the intervention may be related to the fact that it was facilitated on-site, and it was an invitation to stop on a voluntary stance. The aim was to practice and train self-care strategies without adding excessive time-consuming practices and strain. In addition, the sessions were open to all members of the team working at that moment, so people were not marked or stigmatized as someone who specifically needed mental health support.

We asked for adverse effects and three participants reported increased anxiety and dizziness. A recent review about the possibility of harm in mindfulness-based programs concluded that adverse events are no more common in these programs than comparison conditions (exercise or psychotherapy) and may not be attributable to the intervention or are not clinically significant (31).

We provide some hypotheses that we are currently exploring with a qualitative research method in a representative sample. The intervention may be considered as a peer support strategy. Members of the Mental Health Team were in the acute wards with healthcare employees sharing the same reality and all emotions that could arise. This may have helped participants generate a sense of closeness and connectedness which is associated with calmness and helps counteract experiences of stigma and shame (32). In a context where collaborative networks are as critical as they are fragile (because of professionals' deployment or fatigue), this is of great importance. Moreover, the invitation to participate in the sessions could reduce stigma and empower the person who actually connected with their own resources. Delivering the intervention in a group format might also alleviate the sense of loneliness and foster a feeling of “being part of” which has been found to be beneficial (33). Finally, having these mindfulness slots was useful for detecting professionals in situations of special vulnerability. In these cases, we invited them to take care of themselves and contact other members of the Mental Health Team for individual support.

Interventions like the one we describe in this article might constitute a beneficial response to some of the challenges faced when supporting frontline healthcare providers (34, 35). Some of these challenges are: the consideration on the part of managers and the own health professionals that self-care is a luxury and not a need; struggles of healthcare professionals with being in touch with their own feelings, and the recognition of emotions such as fear or anxiety that may produce shame or guilt; asking for help and support can be associated with stigmatization (36); frontline health workers tend to be in “doing mode” during the emergency, having difficulty to take or make little breaks during work time (24).

We cannot generalize the acceptability to all professions. The intervention was well-accepted specifically by nurses and nursing assistants. Doctors had more difficulty to make a pause and some of them reported “not having time,” or “not being interested in this approach.” The question why doctors are not interested or not convinced to participate needs further investigation. In addition, the majority of the participants were women, who have shown to be more empathetic and compassionate than men (37) and maybe more willing to participate in the sessions and contribute filling out the questionnaire. However, up to 70% of people who work in health professions are women according to global data of the 2019 Labor Force Survey in Spain (38), which might explain the high percentage of female participants in our sample. Moreover, we cannot state that this type of intervention could be implemented in settings with no mindfulness experienced professionals. In this study, psychiatrists and clinical psychologists specifically trained in standardized mindfulness programs delivered sessions and supervised the intervention done by professionals with less experience in mindfulness.

The findings of this study are promising and show the feasibility and safety of a brief mindfulness-based intervention to promote healthcare workers' well-being in highly demanding places like ICUs and emergency units. More studies are needed in cross-cultural contexts. The World Health Organization popularized the slogan “no health without mental health” to underscore how important mental health is. We agree with this claim. Furthermore, we assert “no healthcare without self-care.”

## Data Availability Statement

The raw data supporting the conclusions of this article will be made available by the authors, without undue reservation.

## Ethics Statement

The studies involving human participants were reviewed and approved by La Paz University Hospital Research Ethics Committee. Written informed consent for participation was not required for this study in accordance with the national legislation and the institutional requirements.

## Author Contributions

BR-V, ÁP, AM-S, and CB wrote the first draft of the manuscript and reviewed the successive versions of the manuscript. RM, MT, and M-PV-V made relevant contributions to the manuscript. All authors contributed to the implementation of the intervention in an acute setting and approved the final version of the manuscript.

## Conflict of Interest

The authors declare that the research was conducted in the absence of any commercial or financial relationships that could be construed as a potential conflict of interest.
